# Characterisation of precursory seismic activity towards early warning of landslides via semi-supervised learning

**DOI:** 10.1038/s41598-024-84067-y

**Published:** 2025-01-06

**Authors:** David Murray, Lina Stankovic, Vladimir Stankovic, Stella Pytharouli, Adrian White, Ben Dashwood, Jonathan Chambers

**Affiliations:** 1 University of Strathclyde, Glasgow, G1 1XR UK; 2 British Geological Survey, London, UK

**Keywords:** Landslides, Machine learning, Data driven, Natural hazards, Electrical and electronic engineering

## Abstract

This study demonstrates that machine learning from seismograms, obtained from commonly deployed seismometers, can identify the early stages of slope failure in the field. Landslide hazards negatively impact the economy and public through disruption, damage of infrastructure and even loss of life. Triggering factors leading to landslides are broadly understood, typically associated with rainfall, geological conditions and steep topography. However, early warning at slope scale of an imminent landslide is more challenging. Through semi-supervised learning for seismic event detection from continuous seismic recordings over a period of about 10 years, we demonstrate that timely landslide induced displacement prediction is possible, providing the basis for landslide early warning systems. Our proposed methodology detects and characterises seismic precursors to landslide events making use of seismic recordings near an active slow moving earth slide-flow using a semi-supervised Siamese network. This data driven methodology identifies increase in microseismicity, and the change in the frequency spectrum of that microseismicity which identify key stages prior to a failure: ‘rest’, ‘precursor’ and ‘active’. Due to the semi-supervised nature of Siamese networks, the methodology is adaptable to discovering new types of distinct events, making it an ideal solution for precursor detection at new sites.

## Introduction

The growing intensity of landslides is an increasing threat to humans and infrastructure, especially around railways, highways, reservoirs, tailings dams, canals, and densely populated areas. Hence, a deeper understanding of landslides and the ability to predict their occurrence is now more timely than ever. Conventional approaches to landslide monitoring rely heavily on observations of changes on the ground surface (aerial, terrestrial and satellite images and displacement data) assigning levels of risk to certain areas based on local geological and geomorphological features  ^1,2^. There is a large volume of work on studying the kinematics of landslides after failure has occurred as well as risk assessments of sites prone to landslides, i.e., susceptibility assessments, from multi-scale spatial information from field surveys and aerial/satellite data at the catchment-to-regional-scale ^1^. Though subsurface instrumentation to monitor moisture content, acoustic emissions, and displacement via a range of geoelectrical, geotechnical and geodetic methods has shown that there is a relationship between pore pressure and moisture content, particle-particle interactions and indirectly, slope failure ^3–5^, due to sparse sampling when using land surveying technologies, identification of landslide precursors is not possible. Consequently, timely prediction of imminent landslides at field-scale remains a challenging problem.

A favourable alternative would be the use of seismometers to record seismic signals originating from crack formation, propagation or shear within the soil mass. The caveat is that such signals are challenging to analyse due to their small signal-to-noise ratio and are often overlooked or ignored as their analysis is time consuming. For the precursory signals of imminent landslides to be useful, analysis and outcomes need to be as close to real time as possible. The aim of the work reported in this paper is to evaluate the reliability of advanced machine learning for detecting seismic precursory signals originating from shallow landslides in particular, based solely on seismic recordings, and hence provide a cost-effective landslide prediction approach, instead of more resource-intensive indicators such as soil mass displacement measurements.

With the advancements in machine learning, recent years have witnessed a huge expansion of seismic signal analysis from seismometer recordings, with more recent solutions based on very deep neural networks demonstrating good performance in detecting earthquakes, including those triggered by volcanic activity; for example, deep complex neural networks, trained for detecting earthquakes with M$\ge$1.6, such as Earthquake Transformer ^6^, PhaseNet ^7^ and RockNet ^8^. Furthermore, the even more challenging problem of detecting microseismic signals for *low seismic magnitudes* has been gaining traction recently ^9,10^, detecting and classifying tremors and rockfalls from landslides, in addition to larger earthquakes. the adoption of Siamese networks for classification of seismic signals has not been attempted yet, except in^11^, where a Siamese model is used to enhance earthquake picks (detection) from EQT^6^. For recent approaches, using hand-crafted feature generation & selection for classification, Li et al.^12^ provide an up-to-date review, including a breakdown of the most commonly used features and feature importance, as well as highlighting some of the current issues faced by the industry standard approaches using STA/LTA pickers which struggle at detecting events with low spatial and temporal separation another review paper by Li ^13^  highlights newer machine learning approaches. Besides recording rockfalls and other mass movements, seismometers also record tectonic earthquakes, oceanic microseisms, ground and air traffic or other kind of human-induced seismic sources, animal footsteps and rainfall. Hence, the resulting complex seismic wavefield is a mixture of all these sources occurring at different magnitudes and frequencies, making the analysis and interpretation of these seismic records difficult. Furthermore, these detection and classification methods are supervised and hence, “model-constrained”, in that they are trained to detect known seismic classes of events, by learning expected signal patterns, and cannot robustly detect unexplored patterns that could be precursors for understanding the evolution of the subsurface processes.

To overcome constraints of supervised learning approaches, a semi-supervised approach was proposed for seismic signal classification in the frequency domain ^14,15^, detecting 10 earthquakes within a 50km radius from an area known for small and large-scale debris flows, the Rest and Be Thankful (Scotland, (56$\circ$13’15.6”N 4$\circ$50’35.0”W)) , and identifying additional rockfalls and micro-quakes related to landslide processes. Small and medium landslides (around $1.2-1.5 * 10^6 m^3$) are identified, using recordings from just over 100 stations, during the typhoon season in Japan ^16^. The methodology ^16^ can identify previously unknown landslides and requires only minimal assumptions to cluster possible events. However, all the events in^14^ and^16^, are detected only after they occurred and the proposed methods do not identify precursors.

The presence of precursory signals for landslides has only been observed in the laboratory where it was shown that micro-cracks occur prior to a simulated landslide, with the soil being slowly raised to increase the inclination to the point of failure. Just before failure, the rate of micro-cracks increased and the background frequency changes, however due to the scale of the experiment (8 cm soil thickness in a 50 cm square box) the precursor phase is in the order of seconds ^17^. Evidence of precursory signals in the field exists for rock failure^18^ in the shape of the formation and propagation of cracks. Time to failure for rocks under stress has been estimated in the laboratory^19^. Crack formation in soil has been detected^20^ prior to collapse/failure, where it was shown that using short-period seismic arrays and a sufficiently high sampling rate of 1kHz, weak precursory signals that could represent early phases of larger-scale slope failure, can be recorded at source-to-receiver distances of 10 to 43 metres. A sequence of smaller micro-seismic events with very low signal to noise ratio, with increasing frequency, was observed ^21^ about 9 hours preceding the massive 2017 Greenland landslide with 35-51 million m$^3$ of rockfall. Detection of 97% of the precursory signals was only possible through deep scattering with learnable wavelet filter banks and Gaussian mixture models. Note that the study was demonstrated on a single massive landslide.

Despite recent progress and demonstrated potential, there remains a clear gap in the literature on the viability of machine learning-based inference on seismometer recordings only, to identify precursors to landslides and hence predict them, which could make a significant impact on accurate, timely and cost-effective prediction of landslides.

Motivated by the above initial findings, we propose a methodology, based on a semi-supervised Siamese Neural Network, which, from seismic recordings from a single station, demonstrate characterisable “precursory” seismic activity prior to “active” seismic activity during slope movement. The proposed method does not require phase-picking or prior knowledge of source types and uses anchors (representative micro-seismic signals) taken from another landslide with significantly different geological makeup in comparison. In the Results section, we demonstrate consistency of the prediction of three distinct phases of seismic activity, namely “rest”, “precursor” and “active”, that characterise slope movement over a period of about 10 years, cross-referenced against movement data of control points on the ground for validation. In the Discussion section, we characterise the frequency spectrum of these three phases and discuss, in relation to these findings, how landslides can be reliably predicted 2 to 4 weeks prior to occurrence.

## Results

### Hollin hill landslide observatory

**Figure 1:**
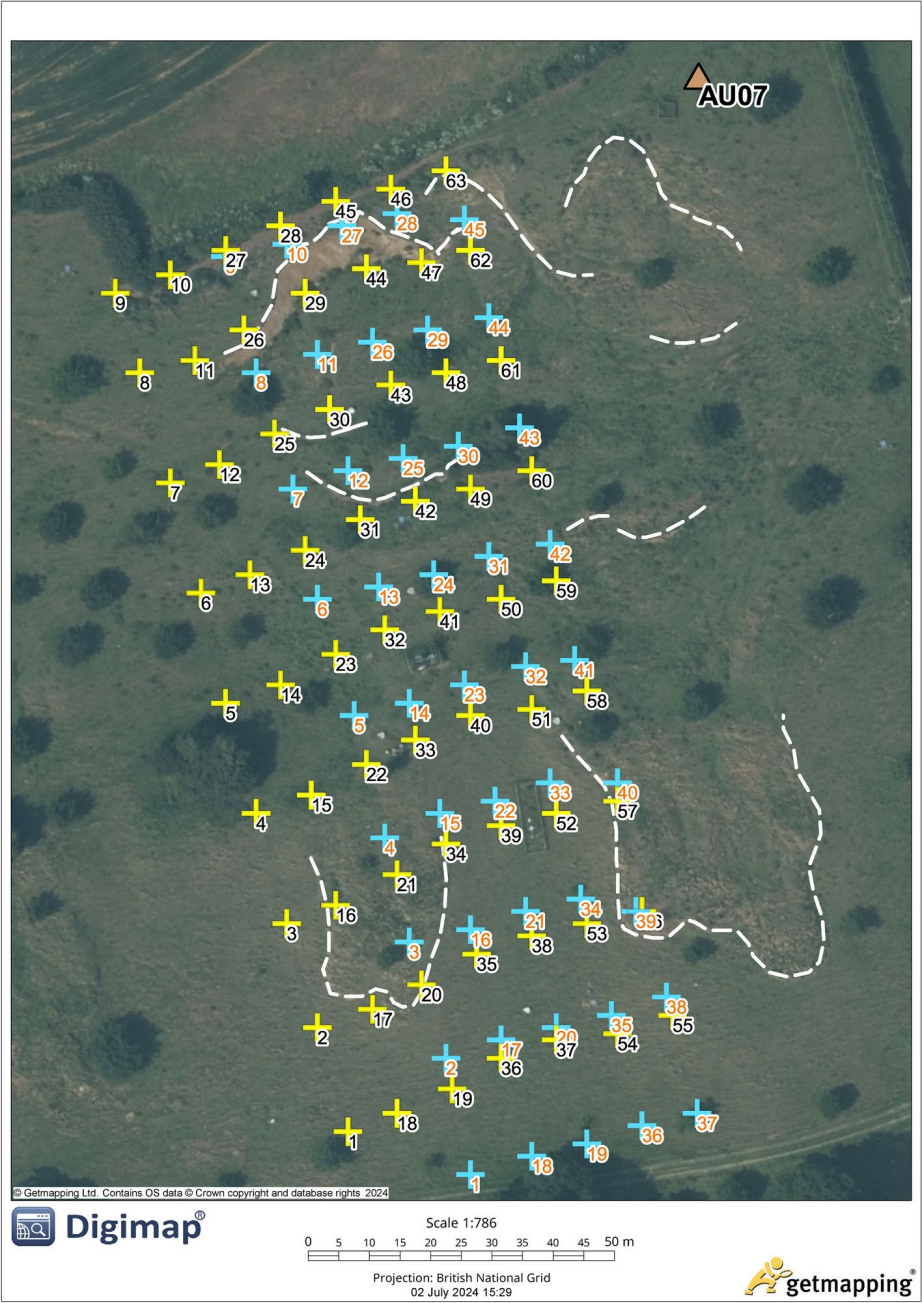
Peg positions, the old peg system is in blue (orange peg numbers) and the new peg system is in yellow (black peg numbers). The seismometer AU07 is identified in the North East of the field. The dashed white lines highlight the flow lobes (East and West), scar traces (located across the middle and upper section), and the backscarp (contained within the borders of the peg systems). The map is orientated due North.

The Hollin Hill Landslide Observatory (HHLO) near Terrington, North Yorkshire, United Kingdom (UK), has been monitored continuously since 2008 with a range of geophysical, geodetic and geotechnical technologies. Due to the high level of ground-truth information provided by ongoing and long-term observations, and regular slope displacements, and in many ways is representative of inland landslides in stiff clay  ^22^, makes it is an ideal site for landslide studies. Hollin Hill is also compared to other slow moving landslides in Lacroix et al. where a large number of site across the globe are discussed including the driving forces^23^. In a more focused comparison limited to Western Europe S.R.Carriere et al. compares the rheological properties of clayey soils in flow-like landslides in which Hollin Hill is shown as comparable to a number of other sites within the European Alps  ^24^. The landslide is a very slow to slow moving composite multiple earth slide-earth flow, and has a mean slope angle of 12$^{\circ }$ and is south facing^25^. The landslide is characterised by rotational slides/failures along the backscarp towards the top of the hill (WMF)) which are considered extremely rapid moving, multiple discrete translational displacements in the mid-slope region (where the WMF meets the Staithes Sandstone Formation to form a boundary) and are generally very slow to moderate in movement, which then transition into a series of earth-flows towards the base of the slope where another boundry is created by the Redcar Mudstone Formation (RMF)^22^ and is classed as extremely slow moving. Prior to 2016 observed movement rates have been as high as 3.5 m/yr^3^.

In this study, we process data from broadband passive seismic monitoring on the site - a CMG-3ESP broadband 3-axis seismometer (station code AU07), which was installed on the 18^th^ of September 2015 and has been operational since. While its main purpose is for monitoring earthquakes as part of the UK’s permanent seismic network (operated and maintained by British Geological Survey, this study explores whether broadband seismic recordings can be used to detect precursors to landslides. Furthermore, for the purposes of cross-referencing precursor findings against recorded slope displacement, displacement data of ground control points was used. The control points were realised by 45 wooden marker pegs for the period 2008-07-30 to 2019-11-08 (old pegs). These were replaced due to degradation with 63 new pegs from 2020-10-20 onwards. The peg positions were determined using Global Navigation Satellite System (GNSS) measurements and readings were taken sporadically mainly at 1 to 3-month intervals with the longest being 6 months. The pegs are arranged in a grid pattern across HHLO. Figure 1 shows the old and new peg locations (last recorded location for old pegs in blue, and initial starting location for new pegs in yellow).

During the monitoring period (2015-onwards) there have been a number of accelerated movements, especially in 2016, 2018, and most recently, at the beginning of 2024. The 2016 slope movement is analysed through morphology-based co-registration, using images captured via UAV monitoring and without reliance on ground control information, showing ^26^ that surface failure of the backscarp appears to have occurred between February and May 2016, indicating an episodic landslide movement characterised by intermittent gravity-driven downslope movements. Images were matched with observations of the peg positions to verify movements. Up to 8.6 m of lateral displacements was observed ^27^ on the easternmost side of the monitoring array with multiple reactivations of the landslide by August 2018. Analysis ^27^ was performed through algorithms for processing (hydro) geophysical datasets, particularly Electrical Resistivity Imaging data along the hillside, in conjunction with the GNSS measurements of the peg positions. The January 2021 movement ^28^, between 18 November 2020 and 19 February 2021, was captured by Sentinel-1 InSAR (Interferometric Synthetic Aperture Radar), also analysed in relation to peg displacement, where it was concluded that average InSAR displacement time series capture the down-slope movement and the seasonal behaviour of the landslide in general.

To summarise, many approaches have been used to capture movement on HHLO via ground based and remote sensing, and have been compared with the measured peg displacements for validation. None of these studies, however, leverage on existing seismic monitoring which is already present on the hill and has complete, continuous, records unlike other approaches which have low temporal granularity, which in turn means that they cannot fully capture smaller intermittent movements as part of the episodic landslide across the slope. Furthermore, the previous studies have only focused on the larger movements on the Eastern lobe or backscarp, given the limitations of remote sensing data. This study captures a fuller picture of slope displacement across the backscarp, Eastern, Centre and Western slopes, narrowing previous descriptions of when events occurred and backing them with observed data from the onsite seismometer. Indeed, our results indicate that the displacement described in 2016 ^27^ is likely to have occurred in late April 2016. The majority of displacement attributed to 2018 is most likely to have occurred in late December 2017 and January & February 2018. Finally, the movement that occurred between Sentinel-1 imaging in 2021 most likely took place in the first two weeks of January.

### Long-term observations of the active site

**Figure 2:**
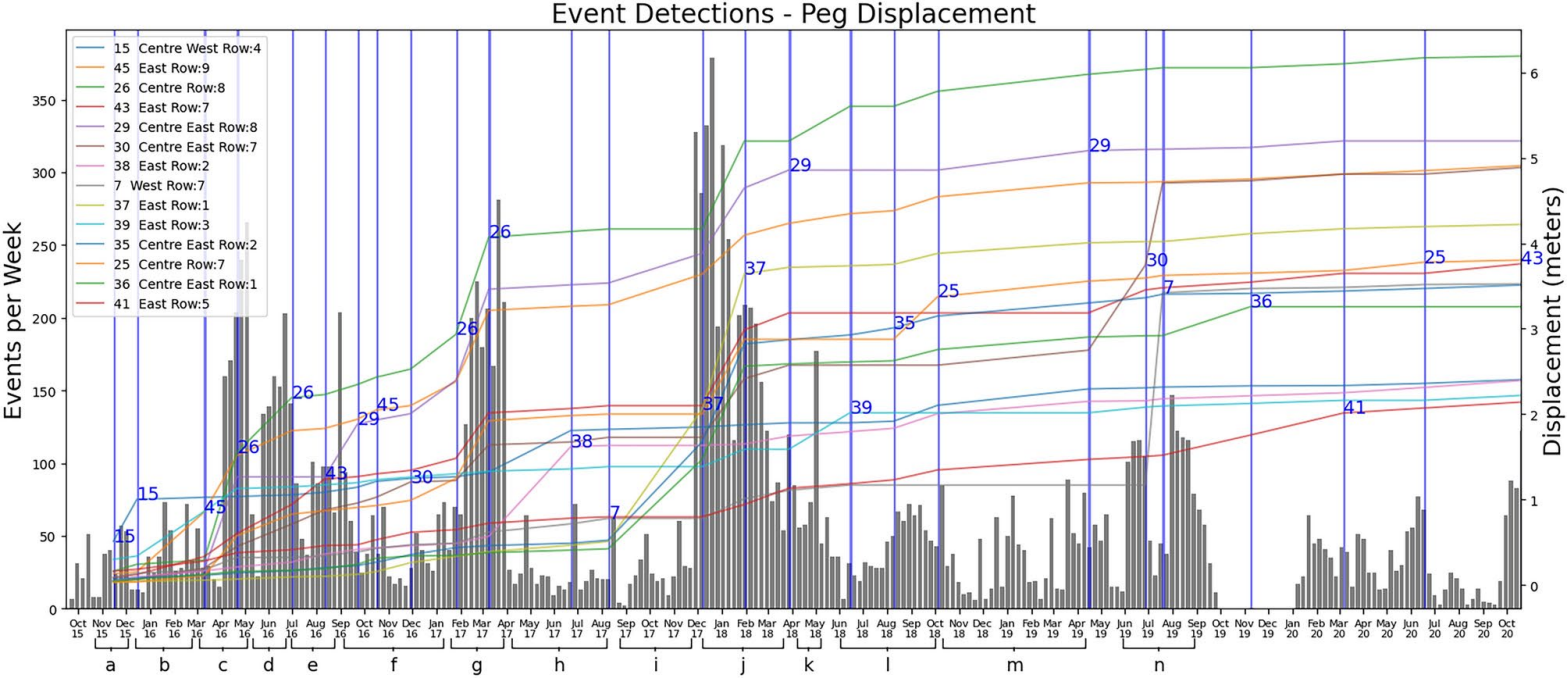
The number of events detected per week. These are overlaid against peg(s) recordings with the greatest displacement over each period (the peg with the largest recorded displacement for the period is shown after the peg epoch (marked with a vertical blue line)). Peg positions corresponding to peg numbers are shown in Fig. 1. Dates on time axis are in month-year format from October 2015.

From September 2015, following the methodology described in Sec. **Methods**, numerous events were detected using solely seismic recordings through the proposed semi-supervised learning. These events are labelled as possible seismic-related events based on their similarity score when compared against randomly selected anchors from the learnt catalogue of micro-seismic signals from another distinct landslide site, namely the complex landslide developed in soft clay-shales in the French Alps and the time-series seismic signals.

Figure 2 shows the total weekly number of detected signals since mid-2015 to October 2020. These weekly bins are overlaid against observed peg displacement (coloured solid lines representing cumulative reading). Pegs were selected based on maximum recorded displacement between recordings. That is, the index number of the peg which recorded the greatest displacement during monitoring appears in blue at the end of said period. We can see that in the lead up to and during the periods where there are large observed peg displacements in mid-2016, early 2017 and early 2018, there are also notable increases in the number of detections per week by the proposed semi-supervised learning. This pattern repeats for the period in mid 2019 in which peg 30 and then peg 7 show a large displacement over a very short period of time. When looking at these larger displacements there is also a trend that the number of detections rises slightly, then has a period where detections fall relative to weekly rate for the previous weeks.

Figure 2, section a: in the last 2 weeks of October 2015 , we see 2 weeks where detections are very low with 8 detections per week. We characterise those as ‘rest’ periods, i.e., periods with very little or no activity. This is followed by a sharp rise to around 40 detections per week for the following two weeks, with a slight drop to 24—we characterise this as the ‘precursor’ stage. Finally, we characterise the peak detections of 57–53 over the next two weeks (the last two in November) as ‘active’ periods, likely where a significant or relatively large displacement occurs in comparison to previous weeks as the landslide is always in some sort of flux. This coincides with the displacement in peg 15 which is located in the lower middle of the hillside on the Eastern edge of the Western flow lobe the lower detections for the amount of movement could be attributed to the distance to the seismometer.

Figure 2, section b: The following weeks (month of December) return to a ‘rest’ period with a detection rate of around 13 per week for the following 3 weeks. From Jan to March 2016, this rest-precursor-active pattern repeats with small peaks and troughs, corresponding to the displacement seen in peg 45 which is located on the Eastern edge of the backscarp failure with over half a meter of movement.

Figure 2, section c: After the first week in March we observe another ‘rest’ period characterised by another drop in detections. This is followed at the start of April by the ‘precursor’ period, characterised by a relatively large increase in detections (from 160 - 240 detections), followed an ‘active’ period, characterised by 266 detections at the end of the first week of May. This sequence of detections is likely highly correlated to the large displacements of more than a meter seen in pegs 26 (the largest displacement) and peg 45 which are both on the top two rows of the monitoring array and located centrally and to the East, respectively just below the backscarp which suffered from a large rotational failure around this time period.

Figure 2, section d: Following this peak, there is a sharp drop in detections for two weeks (‘rest’ period) in the middle of May followed once again by a large increase (‘precursor’ period) followed by the ‘active’ period, with a peak of 203 detections after 4 weeks have passed.

Figure 2, section e: Peg 43 (also located on the upper East of the monitored area) shows the largest displacement which then moves again in the following peg epoch. Despite the lower number of weekly detections we still see a rest followed by an increase although it could be reasonable to assume that the ground is already destabilised and we still observe the steady increase throughout the period moving around 2m over the 5 month period.

Figure 2, section f: The same pattern was observed from September 2016 until January 2017; there is little movement, but still with the occasional ‘active’ and ‘rest’ periods albeit with lower detections in total. This coincides with a slow movement across the entire landslide seen by all pegs displacing slightly.

Figure 2, section g: This then changes after the first week in February as detections double and then rise again. The following 5 weeks consist of relatively high detections as a precursor to backscarp movement and then a peak above 250. This is validated by displacement of peg 26 which is at the centre top of the hillside located below the backscarp and is the best indicator of the backscarp movement.

Figure 2, section h: This is then followed by a large period of rest from the start of April 2017, and the largest displacement during this period is Peg 38 located below the Eastern flow lobe. Based on the number of detections we believe that this movement was likely at the start of the peg epoch at the end of March 2017. Hence even though the granularity of peg recordings is very low during that period, the constant granularity of seismic recordings and the number of seismic-like signals detected, provide more temporal granularity on displacements across the landslide. One of the major displacements which affected the East flow lobe, with 2m displacement, was observed between August 2017 to May 2018.

Figure 2, section i: The last two weeks in August show the lowest number of detections, this is then followed by the pattern of ‘rest’ to ‘precursor’ to ‘active’ periods and back again. In this case however the peak in the first week of November 2017 is then followed by a small drop (60 to 29 to 28) then a huge increase (28 in a week to 328) in the following months. Therefore, the ‘rest’ period of the big displacement observed by peg 37 located below the Eastern flow lobe was likely the first weeks in September 2017. This would then be followed by two small active phases in the last week of September again in the second week of November.

Figure 2, section j: With the high number of detections in the last two weeks before the peg readings (first week of December 2017) we can assume that the landslide started moving significantly around this time: between this reading and the next where peg 37 and 29 are displaced further, the number of detections remains very high with the lowest being 116.

Figure 2, section k: In the last section at the end of April there is a peak which could be responsible for the displacement on peg 39 on the Eastern flow lobe located towards the bottom of the site.

Figure 2, section l: From June 2018 we see initially a very low number of detections in the first week (‘rest’), followed by a marked increase, a small drop and then steady detections throughout July (‘precursor’), , this then jump up again at the beginning of august which coincides with movement in peg 35 and then continues to stay ‘active’ throughout August where peg 25 has the largest displacement but it can be seen that numerous pegs also move during this period as well.

Figure 2, section m: After the first week of October 2018 there is a steady decrease of detected events, with a small spike in detections over the last week in November. There are then a number of peaks in January, late February, and late March. In the 6 and half months between peg readings there is generally a uniform displacement across the site (where displacement is static this is down to missing data for that specific peg). The period of activity which begins in late March is likely the induction of the displacement seen in the April to July readings in 2019 where all pegs have a similar displacement with peg 30 (located centrally in the transistion between rotational and translational failures) being the largest.

Figure 2, section n: The pattern of increased detections can also be seen in other periods throughout the rest of the period monitored via the first peg system, with smaller displacements resulting in smaller peaks and the largest of the movement in June to September 2019 where peg 7 (located on the Western side of the hill between the transition between rotational and translational failures) was displaced around 2 meters (peg 30 with is further displaced is in the same row.

**Figure 3:**
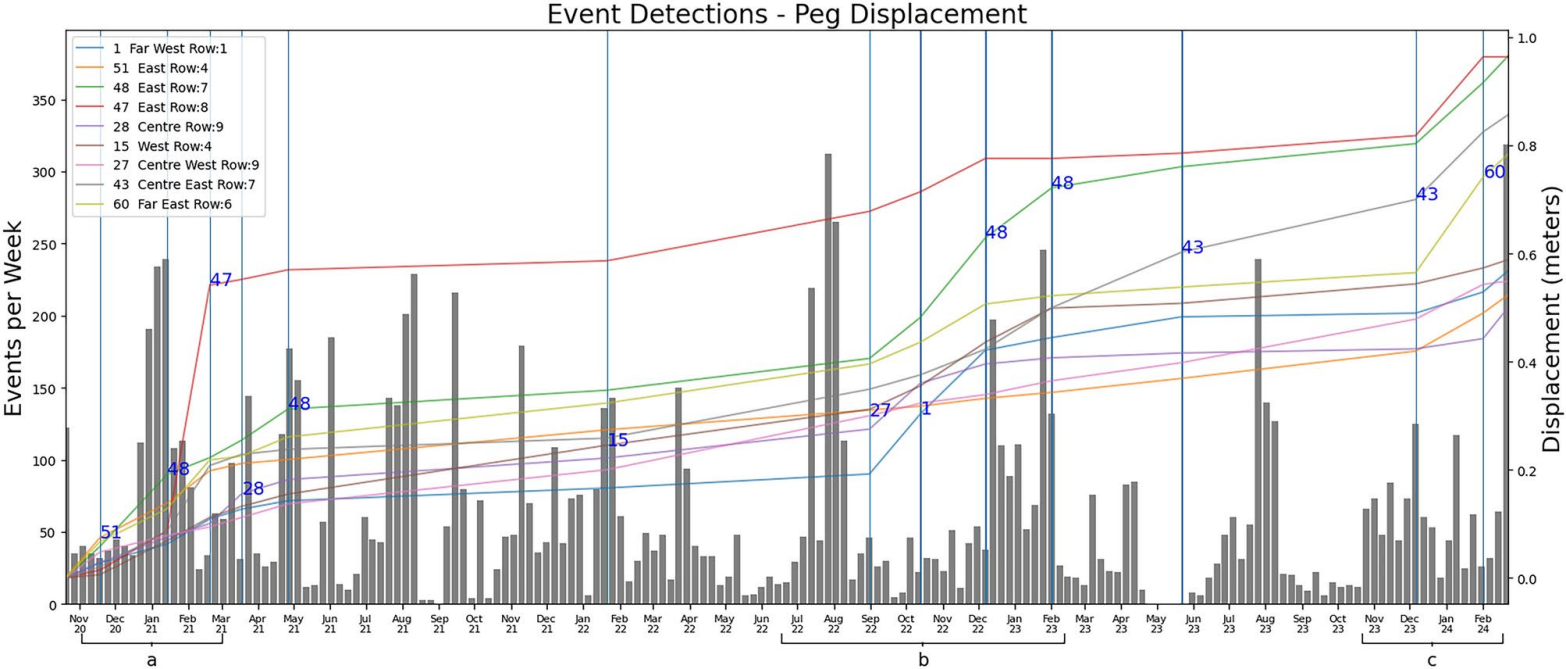
Number of detected signals per week from late 2020. These are overlaid against the new peg array with the greatest displacement over each period.

In late 2020, the new peg system was installed, comprising two additional rows of pegs further to the west of the original ones, with all of the original ones also being replaced, although not at the exactly same location. As such, the cumulative displacement has been reset. During this period the areas of interest are the very beginning of the measurements, November 2020 to March 2021, May 2022 to February 2023 and February 2024.

Figure 3, section a: The largest single displacement happens within the third peg reading period. We observe in the first period (between peg readings) few signals detected, less than 50 per day, as a ‘rest’ period prior to the displacement of 0.25m. Although peg 51 had the maximum displacement, a number of pegs all moved in line with each other. After a surge of about 130 detected in Nov 2020, there is a period of ‘rest’ which is followed by the same precursor ramp up in the two weeks prior to January 2021 and ends in an active peak in the second week of January where peg 47 has the 0.25m displacement. After this, there is another drop to around 24 detections before slowly reaching a lower peak in the second week of March. Overall in this ‘rest’ sequence there are fewer detections and this is reflected in the relatively low displacement seen in this period where peg 28 has the largest displacement of only a few centimetres.

Figure 3, section b: After May 2021, we can observe a series of intermittent ‘rest’, ‘precursor’ and ‘active’ periods culminating in a precursor ramp up from June to mid July in 2022, and again during December 2022 which relate to peg displacements 27 and 48, respectively. The time between peg readings makes the period where peg 27 has the maximum displacement difficult to pin down exact movement, additionally the movement is not sudden as evidenced by the steady rise between displacement from May 2021 through September 2022. However, based on the intensity of detections, the end of July suggests the majority of displacement will have occurred at this time or that the hillside has been destabilised. The rest period at the last two weeks of September 2022 followed by a small active period mid October and then again at the end of December show possible destabilisation times for the displacement associated with pegs 1 and 48. After this period there are then a drop in detection intensity which corresponds to a reduction in displacement deltas across all pegs until December 2023.

Figure 3, section c: Finally, the after a rest period within September and October 2023, detections once again increase during this period peg 43 has been the most displaced being located centrally below the backscarp in the transition between rotational and translational failures. From November 2023 detections rise and fall of detections and finally culminating in a week with over 300 detections coinciding with displacement maximum on peg 60 but will all pegs displacing.

In total, our methodology identified landslide displacements of differing sizes; 24 between 2016 to 2021 and 13 between 2021 and 2024, validated by various peg measurements across the landslide. Of these 37 displacements, only 3 were analysed and documented in detail in prior work^26–28^. However, a number of the remaining displacements, while not specified with the temporal resolution of this work and spatial resolution provided by pegs, can be attributed to the cyclic activity on the hill and will be contained within the larger movement of the events detailed in the prior work of the 2016, 2018 and 2021 documented displacements. The displacements outside of these periods could not be observed due to the limited granularity of peg observations and therefore provide better temporal resolution that can help us better understand the underlying processes involved, culminating in larger landslides and the frequency of smaller displacements.

## Discussion

Our results, through analysis of continuous seismic recordings from one station over a period of 10 years on an actively moving landslide, have consistently distinguished three distinct periods (based on seismic signal detection intensity), namely ‘rest’, ‘precursor’ and ‘active’, that can characterise the stages of displacement. Of particular importance are the precursory periods, whose frequency spectrum can be further characterised in order to predict the onset of displacement, providing temporal and spatial coverage that are generally not captured by displacement pegs alone due to limited and irregular granularity of peg observations. These periods appear to be in the order of 2 to 4 weeks (for larger displacements) based on the intensity of detection and the difference in frequency spectrum in comparison to the periods of maximum detections and lower intensity periods.

**Figure 4:**
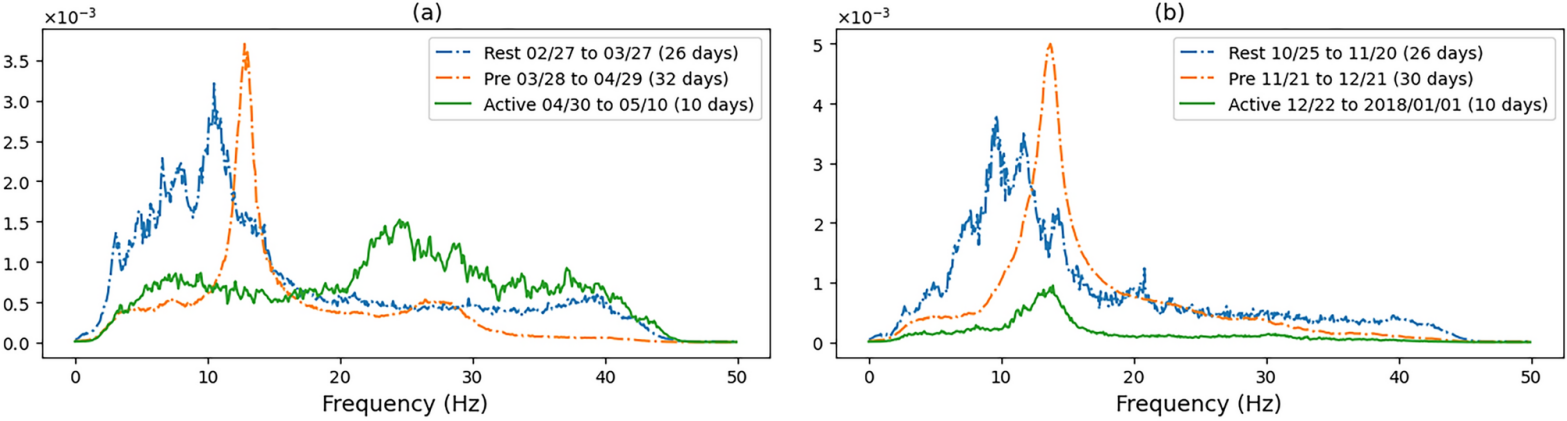
The average frequency components of the signals detected between the dates shown in meters/second as a function of frequency. The title of each plot relates to the displacement period recorded in: (**a**) ^26^, (**b**) ^27^.

**Figure 5:**
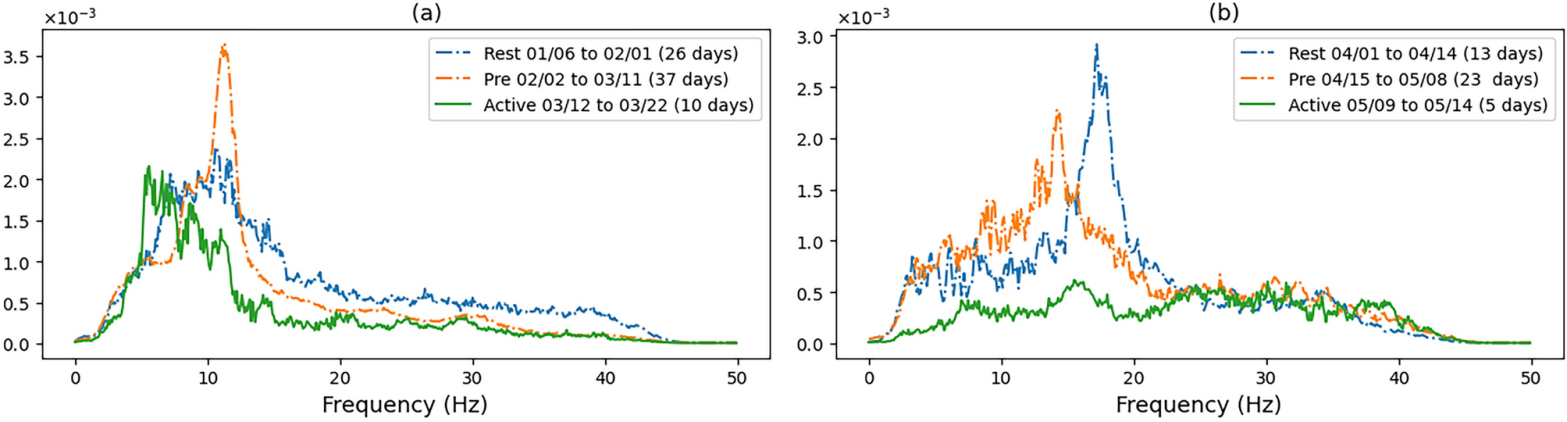
The average frequency components of the signals detected between the dates shown in meters/second as a function of frequency.

In Fig. 4, we show the intensity of all frequency components associated with the three larger displacements on Hollin Hill. First, the frequency spectrum was calculated for each signal using FFT, during the period shown in the legend, and then the FFT values are averaged across all detected signals in the specified period. We can see very distinguishable frequency characteristics of the seismic signals for the three landslide stages. The rest periods tend to have a wider frequency spectrum with main frequency components within 1 to 20 Hz. The change to precursory signals can be seen in all cases and is highly distinct from the resting period signals. The precursory phases tends to have a highly distinct frequency over a narrow band. The active period is typically less distinct in terms of a specific frequency component and covers a larger span of frequencies. This is especially the case with the December 2017 displacement of peg 26 (Fig. 4b), possibly because the active period in this case also contains more precursory signals as the displacement continues after the initial peek in late December through to the start of February. This was also the largest continuous event captured over the monitoring period.

In Fig. 5a we show the spectrum of the March 2017 peg 26 displacement where we can again clearly noticed a distinct precursor component around 12Hz. Figure 5b shows the displacement of peg 39 which is located on the Eastern flow lobe in the lower part of the hill which is primarily a translational failure. The rest period has a different frequency spectrum in comparison to the other backscarp events described. The precursory period however shows the same distinct change with a small narrow peak around 15Hz. The active period has a small peak between the precursory and rest spikes. In this case we believe that the change in the frequency of the events is due to the distance from the seismometer and the change in the event type, the flow lobe being characterised by translational failures, while the backscarp is primarily rotational, additionally the different periods also have shorter durations in comparison to the others described.

Our observations are inline with those of Yfantis^20^ where it was shown that cracks can be detected within a few meters from the seismometers, and failures in clayey soils as small as 2.5 m$^3$ can be detected over the background noise occurring up to 30 meters from the source producing recorded signals in an extended frequency range (20 Hz to 275 Hz). Note however that the seismometer used in ^20^ was sampling at a much higher frequency of 1000Hz compared to the 100Hz sampling frequency at Hollin Hill, and the controlled experiment was conducted on a vertical face in a site which was relatively flat and consisted of unsaturated soil.

**Figure 6:**
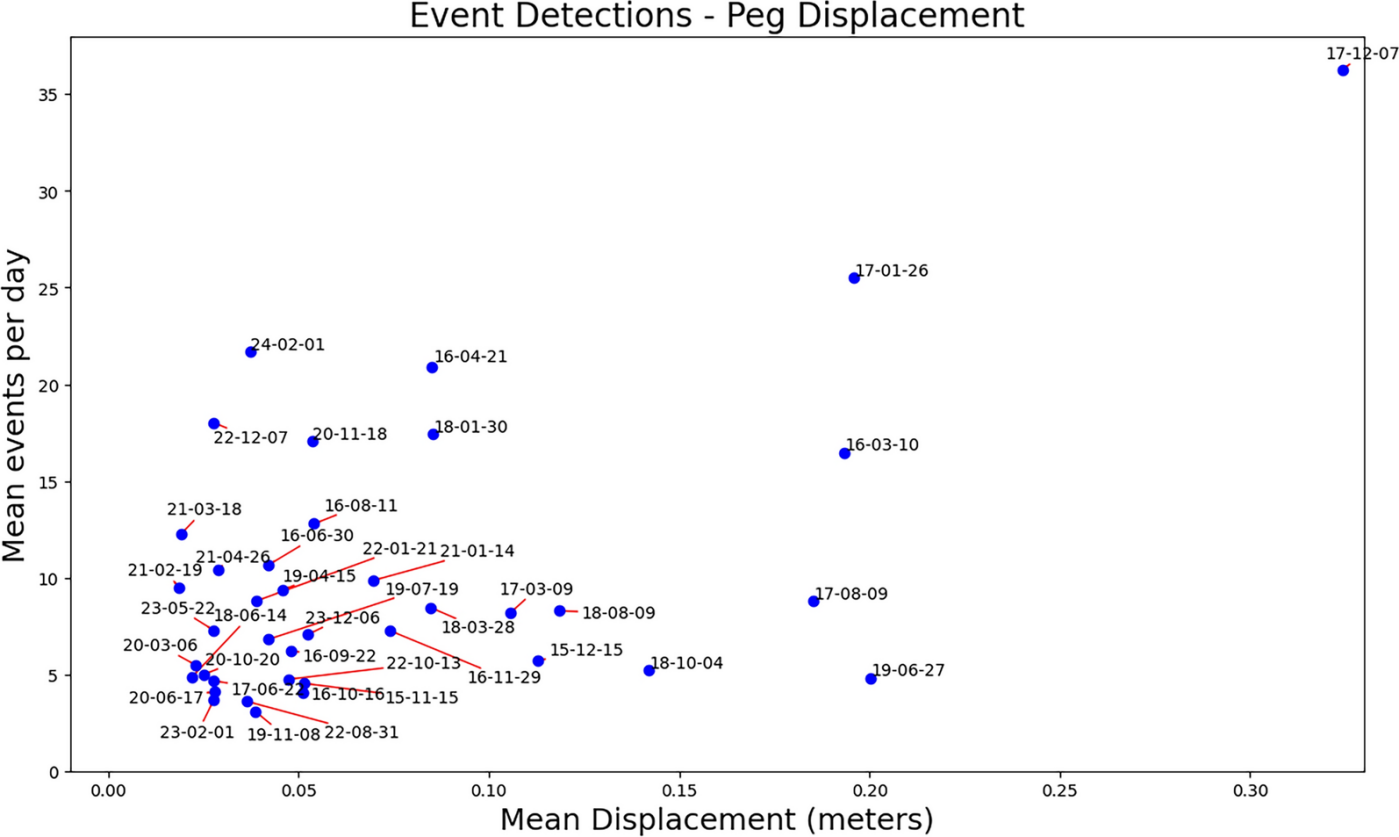
The number of detected events between peg readings against the mean slope displacement between peg readings across all pegs. Peg readings date format ‘yy-mm-dd’.

Furthermore we show that number of events detected is correlated to slope displacement. In Fig. 6 we can see that more events per day results in a higher likeliness for displacement. Many of the readings between 2020 and 2024 however have more events than the expected displacement although this can be attributed to an overall slower movement of the Hollin Hill landslide under the new peg system in comparison to the old peg system, and it can be seen that the key movement periods in 2016 and 2017 are much more highly correlated. The displacement during 2019-06-27 as described in Fig. 2 section n was likely started in the period just prior to the peg measurement.

Therefore, our key contributions are that even with 100Hz seismometer sampling, we observe a noticeable difference in the frequency spectrum between ‘precursory’ and ‘active’ periods, where and precursory frequencies are generally below 15Hz, which together with distinct ramp up in intensity in number of events from a ‘rest’ period, provides a consistent indicator of precursor to displacement, up to 4 weeks before displacement, which tends to coincide with active periods. These results demonstrate that there are key patterns that could be used to predict the onset of displacement within weeks. Additionally, deeper analysis of the frequency spectrum of the precursory and active periods can shed some light on the underlying subsurface processes that cause and explain landslides.

Additionally the semi-supervised detection method is applicable across different landslides and geological conditions, as shown in [geofrontiers], where we show that the detection method detects unlabelled seismic events more effectively than other supervised methods in complex landslide developed in soft clay-shales in the French Alps. The use of the Siamese network allows for the anchor events to be tailored towards specific event types e.g. microquakes or rockfalls. As such transferability to other landslides may be achieved with greater accuracy once an expert has identified a signal of interest. However we expect the relative magnitude and pattern of precursors may vary due to geological conditions and type of landslide. We also plan to explore other types of landslides and geological conditions in future work.

## Methods

**Figure 7:**
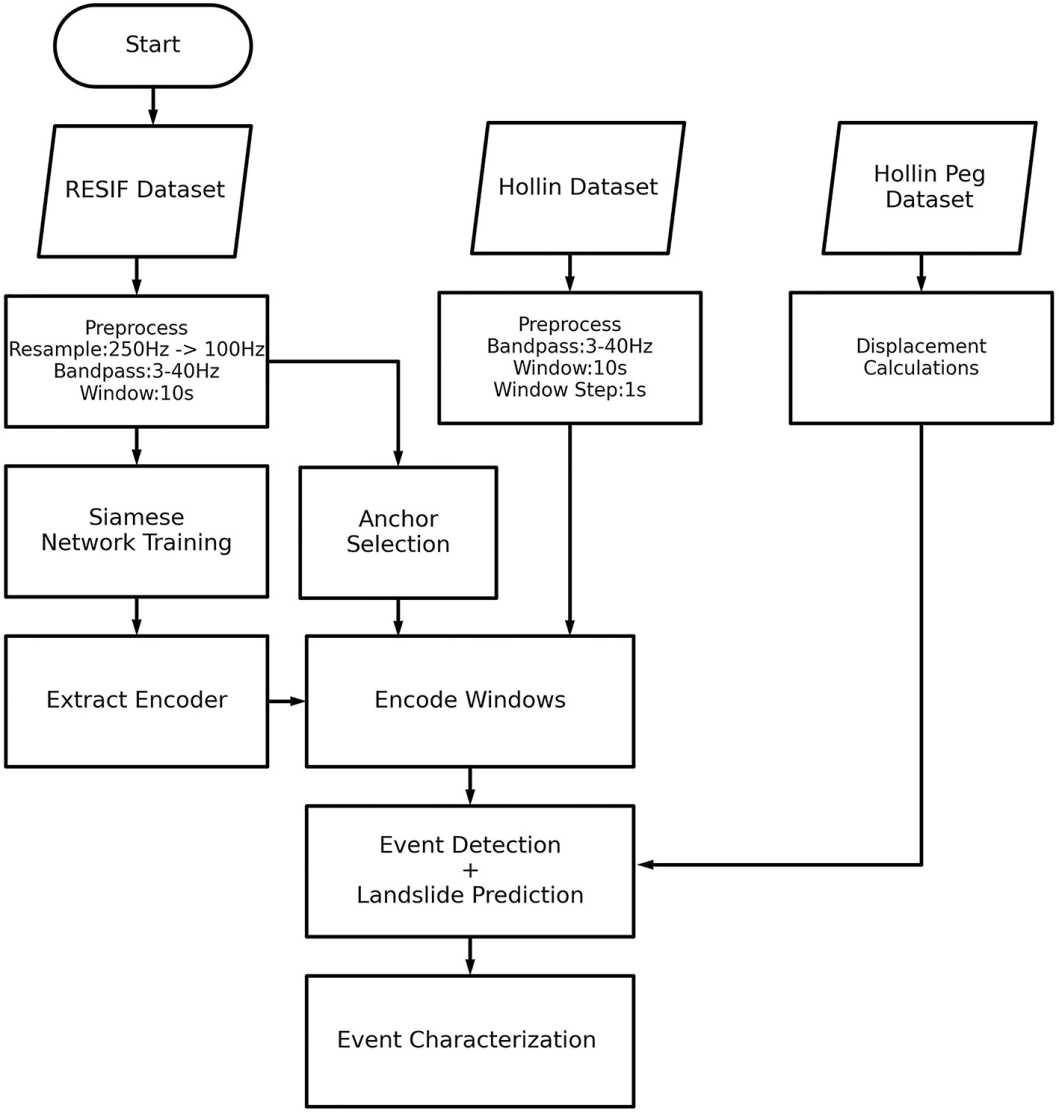
Flow diagram of the proposed method.

The flow of the proposed method used to detected seismic events and predict landslides is shown in Fig. 7. The Siamese network is initially trained using data from the RESIF catalogue^29^. The Super-Sauze landslide is an example of complex landslides developed in soft clay-shales, and has an average slope gradient of 25$^{\circ }$ (double the average of HHLO). Data is gathered by two sensor arrays (SZB & SZC), each with one 3D sensor and three 1D (Z-axis) sensors. The sampling rate is 250Hz and catalogued events (microquake, earthquake (inc. teleseismic), rockfall and anthropogenic noise) are between 0.2 seconds to 105 seconds in length. All event types are used for training to ensure that the network optimises well for a specific anchor. The dataset spans three monitoring periods, 11 October to 19 November 2013, 10 November to 30 November 2014, and 9 June to 15 August 2015. For training, we make use of the MT.SZC station as it has more complete data. Our model uses all three channels (N, E, Z) from the 3D sensor from the SZC array.

Prior to training, we resample the RESIF waveform from 250Hz to 100Hz to match AU07 HHLO seismometer. Then, we use a bandpass filter between 3 and 40Hz which removes the majority of noise which is likely caused by background or anthropogenic sources. The 10-second window is taken from 1 second before the start time of each event within the catalogue.

Training is performed on 70% of the RESIF catalogued data with 30% used for model evaluation. The data is taken in chronological order. Training is performed using 5-fold validation, with a loss patience of 40 and accuracy patience of 80. The initial training rate is 0.0005 and ‘amsgrad’ is set to True, batch size is 256.

Our Siamese network is based on time series input to reduce the overhead of frequency transforms unlike our previous work that uses spectrograms as inputs  ^14,15^. The semi-supervised model’s superior performance on post-displacement catalogued seismic events in comparison with the state of the art is shown in prior work  ^30^. The convolutional part of the network consists of 4 convolutions with drop out on the first 2 and max pooling for the first 3. The filter size is (128, 64, 64, 64), convolution rates are (3, 3, 3, 3), dropout at (0.4, 0.4); all convolutional layers use ‘relu’ as their activation. This is followed by a flatten layer and three dense layers with 128, 64, and 32 units, respectively, which all use ‘sigmoid’ activation. The distance function is calculated using the TensorFlow cosine loss function. The model was trained using TensorFlow 2.10.1. Two events are inputted to the network, and the network is trained to learn the similar and distinguishing patterns between these two input events. Training was completed and for the RESIF catalogue, the following F1 scores were obtained in the four classes; rockfall: 0.81, seism (earthquakes & teleseism): 0.92, quake (microquakes): 0.70 and noise: 0.70. These F1 scores are calculated based on an optimal threshold for all the classes however in our use case the threshold is raised much higher to help reduce the chance of False positives.

After training the network, the encoding section of the network was extracted removing the requirement for two inputs or distance comparison. Five microquake events were selected from the RESIF dataset which contained varying levels of noise, from easily visible in the seismograph to occluded by background noise. These five events were then encoded and saved as five 32-length encoded vectors and will be used as anchors in the event detection.

The HHLO dataset is available in a raw mseed format along with the instrument responses. Utilising the seismometer (station AU07 of the UK Array, BGS seismic network) positioned northeast of the backscarp, our analysis involved examining consecutive 10-second windows from the first installation in 2015 until the present. Firstly, the seismometer response is removed, and a bandpass filter is applied to retain frequencies between 3 and 40Hz, this is done to remove the most likely sources of noise. The Hollin Hill site is also distant from any many roads or well travelled areas therefore noise outside of this frequency band is likely insignificant. Subsequently, 320,571,151 , 10-second (1000 samples) windows (with a 1 second step) are generated for encoding from AU07. Each window is encoded using the trained network into an encoded vector of length 32. Each window encoding is then compared against the 5 anchors (i.e., the five encoded microseismic events from the RESIF dataset).

The calculated distance for every window is saved; the lowest distance recorded was 0.000001. In order to mitigate the number of false positives due to non-landslide sources, we define a threshold that can be calibrated for different sites. The threshold ensures a good trade off between highly similar events and is broad enough to capture variation in the events was the 10th percentile value of the event with the highest minimum, which was 0.02 for HHLO. If the distance between the HHLO recording and the anchors is below a threshold, a potential detection is recorded. If the potential detection is detected by three of more anchors, then an event detection is recorded.

Validation: Precision-recall and other classification metrics cannot be used here since there is no precursor catalogue for HHLO. Hence the semi-supervised approach verified qualitatively against peg movement, used as ground truth. The peg data was recorded in two distinct datasets, both referring to the Ordnance Survey National Grid (OSGB36). The 3D displacement is calculated between each point using:

1 $$\begin{aligned} \Delta d = \sqrt{(x_2 - x_1)^2 + (y_2 - y_1)^2 + (z_2 - z_1)^2}. \end{aligned}$$

The resulting displacements are in meters. Cumulative displacement is calculated by summing recordings chronologically for each peg. The cumulative sum does not cross the old/new dataset boundary as the new pegs were not installed in the exact same location as the previous ones, and doing so would result in a large displacement, as the last reading of the old set and first readings of the new are a day apart. Therefore Figs. 2 and 3 are split between the old and new peg datasets for clarity.

## Data Availability

The Holin Hill seismometer and peg data that support the findings of this study are available from BGS but restrictions apply to the availability of these data, which were used under agreement for the current study, and so are not publicly available. Data are however available from the authors (E.g. Corresponding author) upon reasonable request and with permission of BGS.The Super-Sauze data that support the findings of this study are available from RESIF but restrictions apply to the availability of these data, which were used under agreement for the current study, and so are not publicly available. Data are however available from the authors (E.g. Corresponding author) upon reasonable request and with permission of RESIF.

## Code availability

Code will be made available on https://github.com/David-Murray/TimeSeries-Siamese.
